# Descriptive study of 192 adults with speech and language disturbances

**DOI:** 10.1590/S1516-31802002000600003

**Published:** 2002-11-01

**Authors:** Letícia Lessa Mansur, Márcia Radanovic, Danielle Rüegg, Lúcia Iracema Zanotto de Mendonça, Milberto Scaff

**Keywords:** Aphasia, Etiology, Clinical features, Lesion, Site, Neurolinguistics, Afasia, Etiologia, Quadro, Clínico, Localização, Neurolingüística

## Abstract

**CONTEXT:**

Aphasia is a very disabling condition caused by neurological diseases. In Brazil, we have little data on the profile of aphasics treated in rehabilitation centers.

**OBJECTIVE:**

To present a descriptive study of 192 patients, providing a reference sample of speech and language disturbances among Brazilians.

**DESIGN:**

Retrospective study.

**SETTING:**

Speech Pathology Unit linked to the Neurology Division of the Hospital das Clínicas of the Faculdade de Medicina da Universidade de São Paulo.

**SAMPLE:**

All patients (192) referred to our Speech Pathology service from 1995 to 2000.

**PROCEDURES:**

We collected data relating to demographic variables, etiology, language evaluation (functional evaluation, Boston Diagnostic Aphasia Examination, Boston Naming and Token Test), and neuroimaging studies.

**MAIN MEASUREMENTS:**

The results obtained in language tests and the clinical and neuroimaging data were organized and classified. Seventy aphasics were chosen for constructing a profile. Fourteen subjects with left single-lobe dysfunction were analyzed in detail. Seventeen aphasics were compared with 17 normal subjects, all performing the Token Test.

**RESULTS:**

One hundred subjects (52%) were men and 92 (48%) women. Their education varied from 0 to 16 years (average: 6.5; standard deviation: 4.53). We identified the lesion sites in 104 patients: 89% in the left hemisphere and 58% due to stroke. The incidence of aphasia was 70%; dysarthria and apraxia, 6%; functional alterations in communication, 17%; and 7% were normal. Statistically significant differences appeared when comparing the subgroup to controls in the Token Test.

**CONCLUSIONS:**

We believe that this sample contributes to a better understanding of neurological patients with speech and language disturbances and may be useful as a reference for health professionals involved in the rehabilitation of such disorders.

## RESUMO

**CONTEXTO:**

Afasia é uma seqüela muito incapacitante, causada por doenças neuroló-gicas. No Brasil existem poucos dados sobre o perfil dos afásicos atendidos em centros de reabilitação.

**OBJETIVO:**

Apresentar um estudo descritivo de 192 pacientes, fornecendo amostra de refe-rência de problemas de fala e linguagem em brasileiros.

**TIPO DE ESTUDO:**

Retrospectivo.

**LOCAL:**

Ambulatório de Fonoaudiologia da Clínica Neurológica do Hospital das Clínicas da Faculdade de Medicina da Universidade de São Paulo.

**PARTICIPANTES:**

Todos os pacientes (192) consultados em nosso serviço de Fonoaudiologia entre 1995 e 2000.

**PROCEDIMENTO:**

Coletamos dados referentes às variáveis demográficas, etiologia, avali- ação de linguagem (funcional, Testes Boston para Diagnóstico de Afasia, Nomeação de Boston e Token), e estudos de neuroimagem.

**VARIÁVEIS ESTUDADAS:**

Resultados obtidos nos testes de linguagem, dados clínicos e de neuroimagem foram organizados e classificados.

Setenta afásicos foram escolhidos para compor um perfil. Quatorze sujeitos com lesão de um único lobo foram analisados detalhadamente. Dezessete afásicos foram comparados com 17 controles na execução do Teste Token.

**RESULTADOS:**

100 sujeitos (52%) eram ho-mens e 92 (48%), mulheres. O grau de esco-laridade variou de 0 até 16 anos (média: 6,5; desvio-padrão: 4,53). Os locais de lesão fo-ram identificados em 104 pacientes, 89% em hemisfério esquerdo, 58% devidos a doença cerebrovascular. A incidência de afasia foi de 70%; disartria e apraxia, 6%; alterações fun-cionais de comunicação, 17%; 7% eram normais. No subgrupo comparado a controles no Teste Token, houve diferenças estatistica-mente significantes.

**CONCLUSÕES:**

Acreditamos que esta amostra contribua para uma melhor compreensão de pacientes neurológicos com alterações de fala e linguagem e pode ser útil como referência para profissionais de saúde envolvidos na re-abilitação de tais desordens.

## INTRODUCTION

Among the possible sequelae generated by central nervous system lesions, language and speech alterations are highlighted by the potential functional impairment that may result. Aphasia brings great suffering to the patient, relatives and caregivers. It is estimated that the incidence of aphasia caused by stroke is between 21% and 38%.^1,2^ It is widely known that complete spontaneous recovery from a brain lesion is the exception rather than the rule. Efficient rehabilitation strategies are necessary, in which detailed knowledge of the target population is fundamental. Our objective was to contribute to this knowledge through the description of adults with speech and/or language disturbances treated at a Speech Pathology Unit linked to the Neurology Department of a tertiary-level university hospital (Hospital das Clínicas of the Faculdade de Medicina da Universidade de São Paulo) from 1995 to 2000.

## METHODS

We present a retrospective study of 192 subjects with speech or language alterations. These were attended to via the outpatient service of the Speech Pathology Unit during the five-year period from 1995 to 2000. The patients were indicated: a) by the Speech Pathology team after their evaluation and diagnosis while interned in the Neurology Service, b) by neurologists and geriatricians from other outpatient clinics of the same hospital and c) by graduate students from a teaching and research facility linked to the Speech Pathology Department. Since this is a tertiary university hospital, spontaneous seeking of attendance is almost non-existent in our service. The age of the patients varied from 11 to 83 years (average 51.4 years; standard deviation 17.7 years). Patients were evaluated using formal language tests, including a functional evaluation through closed questions, the Boston Diagnostic Aphasia Examination (BDAE), the Boston Naming Test (BNT)^3^ and the Token Test.^4^ From the total sample, 185 (96.3%) performed the BDAE, 135 (70.3%) performed the BNT, while 69 (35.9%) performed the Token Test. The time interval between the onset of symptoms and the evaluation was extremely variable, ranging from 1 month to 8 years.

Most aphasics present deterioration in word finding, albeit mild. For this reason, we adopted below-normal performances for naming and verbal fluency tests, as the criteria for aphasia diagnosis, thus resulting in a sample of 133 patients (seven patients who did not perform the Boston and 52 with normal scores in the abovementioned tests were excluded). Fourteen subjects with vascular lesions restricted to a single lobe (3 frontal, 4 parietal and 7 temporal) in the left hemisphere were selected to undergo a more detailed analysis of aphasia symptoms. Seventeen aphasic subjects with vascular lesions in the left hemisphere were selected for comparison with 17 normal subjects, all performing the Token Test. The latter were not subdivided according to the specific lesion site, so as to make statistical analysis possible. The data were analyzed utilizing the Mann-Whitney nonparametric method. We considered a value of p < 0.05 as statistically significant. The statistical analysis was accomplished by using the GraphPad InStat® version 3.05 software.

## RESULTS

**Figure 1:**
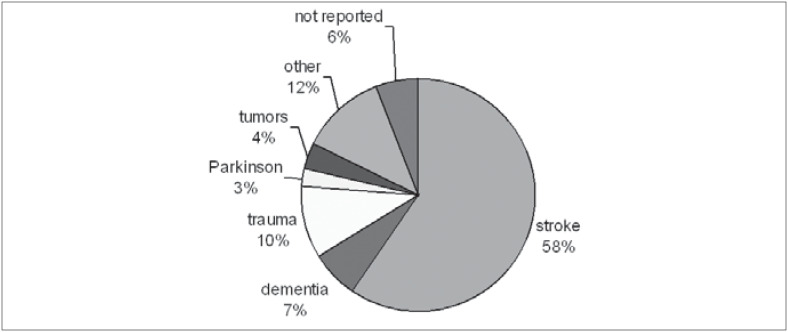
Etiology for the development of speech and language disturbances (n = 192).

**Figure 2:**
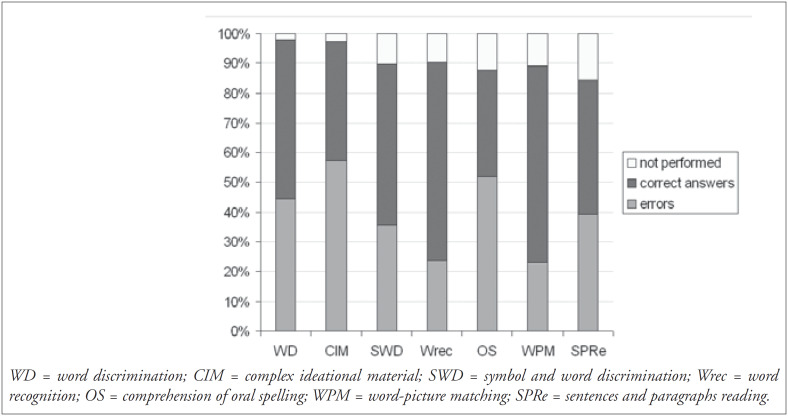
Performance in oral and reading comprehension tests (n = 185).

**Figure 3:**
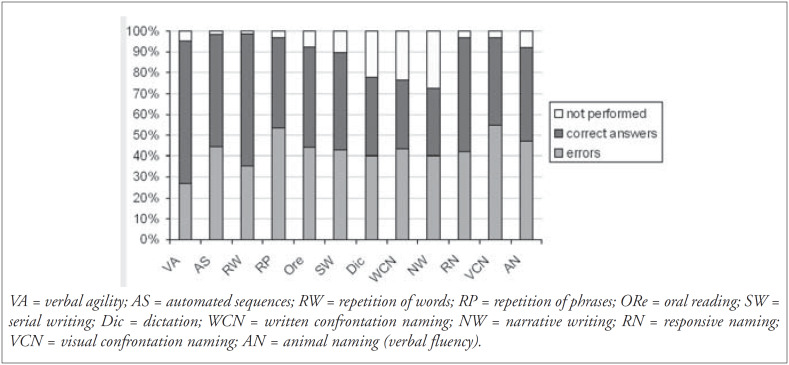
Performance in production (oral and written) and naming tests (n = 185).

In the gender distribution, 100 subjects (52%) were men and 92 (48%) women. Regarding education, we found a variation ranging from 0 to 16 years (average: 6.5 years; standard deviation: 4.53 years). Preferential handedness was exhibited by 124 patients, with 119 (96%) being right-handed. The etiology for the development of speech and language disturbances found in the sample is shown in Figure 1. The frequency of symptoms obtained for the 185 patients who performed the BDAE was analyzed and the profile obtained is shown in Figures 2 and 3.

**Figure 4:**
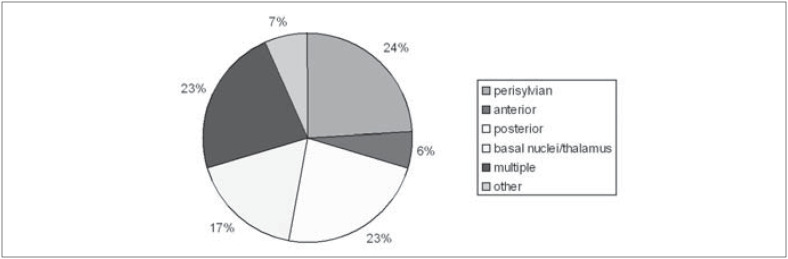
Distribution of lesion sites in 104 cases.

From the cases with focal lesions, it was possible to determine the specific lesion sites in 104 (from neuroimaging descriptions). Their distribution is demonstrated in Figure 4. The lesions were grouped as "perisylvian" (involving the whole language area), "anterior" and "posterior" (relative to the Sylvian fissure), subcortical (basal nuclei and thalamus), "multiple" and "other" (cerebellar and brain stem lesions). Most lesions were situated in the left hemisphere (89%), and there was a total of 11 cases with lesion in the right hemisphere only: 2 thalamic, 1 temporal, 1 frontal, 1 parietal, 3 frontotemporal, 2 frontotemporal-parietal, 1 temporoparietal-occipital and 1 of basal nuclei.

The incidence of aphasics in this sample was 70% (133/192). The remaining 30% consisted of patients with speech motor disturbances such as dysarthria and apraxia (6%) and functional (non-aphasic) communication alterations. In 7% of the subjects the scores were normal. In order to build a performance profile for aphasics from the various Boston subtests, similar to those existing for English speakers,^3^ we chose a sample of 70 subjects with proven left hemispheric lesion from the 133 aphasics mentioned above (Table 1). The diagnosis of aphasia was based on the cutoff scores derived from a Brazilian sample.^5^

**Table 1:** Performance of a sample of aphasic patients in the Boston Diagnostic Aphasia Examination, with mean, standard deviation and range of scores; the first column shows the number of patients with disturbance in each subtest (from a total sample of 70)

SUBTEST	N	RANGE	MEAN	STANDARD DEVIATION
Word discrimination	36	0 – 72	54.6	17.7
Body-part identification	52	0 - 18	11.3	5.2
Commands	36	0 – 11	6.9	4
Complex ideational material	48	0 – 12	6	3.2
Nonverbal agility	18	0 - 12	6.5	3.9
Verbal agility	19	0 - 14	9.2	5.2
Automated sequences	34	0 – 8	5.2	3
Reciting/singing/rhythm	15	0-2	1.2	0.8
Repetition of words	35	0 – 10	7	3.6
High-probability phrase repetition	41	0 – 8	4	3.3
Low-probability phrase repetition	46	0 – 8	3.3	3.1
Word reading	45	0 – 30	17.5	11.7
Responsive naming	38	0 – 27	18.4	10.2
Visual confrontation naming	50	0 - 114	62.1	42.6
Animal naming (Fluency)	38	0 - 26	6.6	5.9
Oral sentence reading	44	0 - 10	4.2	4.3
Symbol and word discrimination	27	0 - 10	7.9	2.9
Word recognition	27	0 - 8	6.2	2.3
Comprehension of oral spelling	43	0 - 8	1.9	2.7
Word-picture matching	24	0 - 10	6.7	3.7
Reading sentences and paragraphs	32	0 – 10	5.7	3.6
Writing mechanics	23	0 – 5	3.3	1.5
Serial writing	34	0 – 45	30.6	15
Primer-level dictation	32	0 - 15	9.3	5.2
Spelling to dictation	35	0 – 10	3.5	3.6
Written confrontation naming	35	0- 10	4.5	4.1
Narrative writing	32	0 – 5	1.9	1.8
Sentences to dictation	34	0 - 12	4.7	5

Upon analysis of the subgroup with lesions in the left hemisphere that performed the Token Test, we observed statistically significant differences in the performance (from the second part onwards), when compared to the control group (Table 2).

**Table 2:** Scoring of patients and controls in the Token Test

Group	part 1		part 2		part 3		part 4		part 5	
	n = 10		n = 6		n = 10		n = 10		n = 21	
	M	SD	M	SD	M	SD	M	SD	M	SD
Stroke	8.35	3	3.3	2.1	5	3.1	2.6	3	4.3	4.75
Controls	9	2.7	5.9	0.5	9.5	1.9	8.9	1.8	17.6	4
*p* value	Ns		< 0.0001	< 0.0001	< 0.0001		< 0.0001			

M = mean; SD = standard deviation; ns = non-significant.

## DISCUSSION

Based on the results obtained for the 185 patients submitted to Boston evaluation, we now present a brief analysis of the symptoms most frequently found in oral and reading comprehension, oral and written production, and naming.

With regard to the oral comprehension subitems (Table 1), we noted that the greatest number of errors was in body-part identification and complex ideational material. Errors in the body-part identification task related to specific regions such as some fingers that are less frequently named. The performance in the complex ideational material task requires integrity of functions varying from the most elementary word comprehension to working memory (necessary for the accomplishment of syntactic analysis), as well as discourse and text comprehension (right hemisphere function). This increases the number of possible sites favoring the appearance of such symptoms.

The analysis of repetition alterations showed that aphasia with repetition disturbances (100/133) was three times more frequent than aphasia without repetition disturbances (75% versus 25%, respectively) (Figure 3). We believe that this finding can be partly explained by the high incidence of stroke as the etiology factor (58% or 111 cases), with perisylvian lesions (in the middle cerebral artery territory) being very common (Figure 4).

The reading comprehension tests (Figure 2) revealed that the patients’ performance was worse in the oral spelling task. Even among normal subjects, this task represents a cognitive requirement that is uncommon for Brazilians, since Portuguese is a language in which there is a high degree of phoneme-grapheme correspondence. In the comprehension of sentences and paragraphs task, it was observed that the proportion of subjects who performed well (83/156 or 53.2%) was greater than in the analogous test of complex ideational material (74/180 or 41%). Written material can be reread, and thus is not as strongly dependant on the temporal processing of information as spoken language is. The same effect had an influence, albeit more discreetly, when the repetition and oral reading tests were compared (Figure 3).

In the written production tests (Figure 3), the distribution of errors was fairly homogenous. Nonetheless, these were the tests with the highest level of non-performance, especially due to low education level and the cooccurrence of right hemiparesis in subjects with left hemispheric lesions.

In the naming subtests (Figure 3), patients performed better in responsive naming tasks than in the visual confrontation naming task. The responsive naming task allows a degree of verbal-semantic association, since the questions contain a related word. In our sample we found 52 subjects who presented normal performance in the responsive naming, visual confrontation naming and verbal fluency tasks. Of these, 48 were submitted to the BNT, in which only 4 (8.3%) obtained a score below normal. This data suggests that the greatest importance of this test is in the characterization of anomia (through the qualitative analysis of errors) rather than in improving the sensitivity of the diagnosis when compared to the naming subtests of the Boston tests, as Goodglass and Kaplan^3^ have already recognized.

The intention of the Token Test authors was to devise a test that would create a totally artificial situation, in which the tested subject could not base his answers on contextual clues or on his habitual speech outlines so as to improve his performance.^4^ We found that from the second part onwards there was a difference in the results of the Token test when comparing brain-damaged individuals and normal ones (Table 2), exactly when the tasks depended on a more specific solicitation of language comprehension and working memory.

The high incidence of aphasics in this sample (70%) was due to the fact that it represented the attendance provided by a group of speech therapists linked to a university service, in which the complexity of cases is greater. The remaining 30% consisted of patients with speech motor disturbances such as dysarthria and apraxia (6%), and functional communication alterations (non-aphasic) such as discourse and paralinguistic disabilities or working memory deficits, related to right-hemisphere, frontal or bilateral dysfunction (17%). In 7% of the subjects the scores were normal. We believe that the profiles shown in Tables 1 and 2 may be useful in classifying the severity of aphasia in Brazilian patients.

Due to factors related to lesion extension, etiology, neural plasticity, previous language competence and the interaction between language and other cognitive functions, many patients fail to fit within recognized typed syndromes. In such cases, the attempt to reduce the patient's symptomatology to a typical pattern can harm the overall view of the real communication problem and compromise the efficiency of speech therapy. In order to illustrate this discussion, we selected some cases with single and circumscribed lesions in the left hemisphere. The discussion is based on the average performance of patients from each group, whose lesions are not necessarily equivalent in size. Occipital lesions were not considered because they are not very likely to cause primary language disturbances.

Frontal region lesions immediately bring to mind classical “expression aphasia”. In fact, grammar and syntax alterations, impoverishment of spontaneous speech and reduction in verbal fluency tests can be found even in lesions that spare Broca's area.^6,7^ In our sample, we found three subjects with frontal lesions and severe problems in the oral comprehension tasks, which were associated with the poor performance already expected in production tests. These patients presented an interesting combination of cognitive symptoms, in which the presence of other frontal symptoms (lack of initiative and attention and working memory deficits, associated with difficulties in strategy choice and maintaining task execution) led to language dysfunction that surpassed what would be expected if we were only to consider the classical language area disturbances. Thus, working memory deficits can interfere with syntactic comprehension and repetition tasks^8-10^ and attention deficits and disturbances in strategy elaboration can also interfere in the results of comprehension tests.^11^ This fact is particularly important in the evaluation of patients with frontal lesions, where central executive functions or behavioral alterations can largely and diffusely compromise overall performance in language tests. Occasionally, supplementary patient evaluation via neuropsychological examination or non-verbal tests is necessary to allow the results obtained in the formal language testing to be interpreted adequately.

In four cases of purely parietal lesions, we noted milder symptomatology in which the characteristics related to reading and writing predominated. In fact, the storage of sensorymotor engrams necessary for writing production and perception is attributed to the left parietal lobe.^12^ In the cases selected for analysis, we did not observe huge repetition alterations or anomia, as would be expected in angular and supramarginal gyri lesions. It is interesting to note the constant difficulties in identifying body parts, which is compatible with the body image representation function executed by the parietal region.

In the purely temporal lesions, we found that language was more severely compromised than in the parietal lesions, with greater involvement of comprehension, naming, reading and writing tasks. It is interesting to note the similarity in performance profile between temporal and frontal lesions. In fact, in the criteria for classical aphasia classification, sometimes the main aspects that permit differentiation between Wernicke and Broca aphasia are fluency and the occurrence and type of paraphasia, since other aspects can be compromised to varying degrees, with the overlapping of symptoms.^3,13^ When some degree of malfunction in frontal executive tasks is added, the classification boundaries can become tenuous. It is worth noting that the differentiation between anterior and posterior language disturbances is more consistently achieved when the detailed aspects of linguistic processing derived from cognitive neuropsychology are studied.

## CONCLUSION

Descriptive and epidemiological studies on the characteristics and behavior of Brazilian people are scarce in the field of neuropsychology. More often than not, clinicians and researchers have to rely on foreign parameters when classifying and working with Brazilian patients. We believe that this description contributes to a better understanding of our neurological patients with speech and language disturbances and it may be useful as a reference for health professionals involved in the rehabilitation of such disorders.

Identification of alterations at various linguistic levels and in comprehension and production processes does not mean that aphasics’ difficulties are restricted to the field of linguistics. The cognitive demands of tasks with varying degrees of complexity makes it clear that cognitive processing has a strong basis in language, and that aphasia can cause extreme difficulties in tasks involving uncertain contents or that have complex organization and integration in linguistic terms. This integrated view of language and other cognitive abilities is recent and has been recognized by neurolinguists who have studied aphasia in many languages.^14^
